# Validity of the global physical activity questionnaire (GPAQ) in Bangladesh

**DOI:** 10.1186/s12889-017-4666-0

**Published:** 2017-08-10

**Authors:** Shirin Jahan Mumu, Liaquat Ali, Anthony Barnett, Dafna Merom

**Affiliations:** 1 School of Science & Health, Western Sydney University, Sydney, Australia; 2 Dept of Epidemiology, Bangladesh University of Health Sciences (BUHS), Dhaka, Bangladesh; 3 Dept of Biochemistry & Cell Biology, BUHS, Dhaka, Bangladesh; 4 Institute for Health & Ageing, Australian Catholic University, Melbourne, Australia

**Keywords:** Global Physical Activity Questionnaire, GPAQ, Validity, Physical activity questionnaire, Accelerometer, Bangladesh

## Abstract

**Background:**

Feasible and cost-effective as well as population specific instruments for monitoring physical activity (PA) levels are needed for the management and prevention of non-communicable diseases. The WHO-endorsed Global Physical Activity Questionnaire (GPAQ) has been widely used in developing countries, but the evidence base for its validity, particularly for rural populations, is still limited. The aim of the study was to validate GPAQ among rural and urban residents in Bangladesh.

**Methods:**

A total of 162 healthy participants of both genders aged 18–60 years were recruited from Satia village (*n* = 97) and Dhaka City (*n* = 65). Participants were invited to take part in the study and were asked to wear an accelerometer (GT3X) for 7 days, after which they were invited to answer the GPAQ in a face to face interview.

**Results:**

Valid accelerometer data (i.e., ≥10 h of wear times over ≥3 days) were received from 155 participants (rural = 94, urban = 61). The mean age was 35 (SD = ±9) years, 55% were females and 19% of the participants had no schooling, which was higher in the rural area (21% vs 17%). The mean ± SD steps/day was 9998 ± 3936 (8658 ± 2788 and 12,063 ± 4534 for rural and urban respectively, *p* = 0.0001) and the mean ± SD daily moderate-to-vigorous physical activity (MVPA) was 58 ± 30 min (51 ± 26 for rural and 69 ± 34 for the urban, *p* = 0.001) for accelerometer. In case of GPAQ, rural residents reported significantly higher moderate work related PA (MET-minutes/week: 600 vs. 360 *p* = 0.02). Spearman correlation coefficients between GPAQ total MVPA MET-min/day and accelerometer MVPA min/day, counts per minute (CPM) or steps counts/day were acceptable for urban residents (rho: 0.46, 0.55 and 0.63, respectively; *p* < 0.01) but poor for rural residents. The overall correlation between the GPAQ and accelerometer for sitting was low (rho: 0.23; *p* < 0.001). GPAQ-Accelerometer correlation for MVPA was higher for females (rho: 0.42), ≤35 age group (rho: 0.31) and those with higher education attainment (rho: 0.48). The Bland-Altman plots illustrated bias towards over estimation of GPAQ MVPA with increased activity levels for urban and rural residents.

**Conclusion:**

GPAQ is an acceptable measure for physical activity surveillance in Bangladesh particularly for urban residents, women and people with high education. Given waist worn accelerometers do not capture the typical PA in rural context, further study using a physical activity diary and a combination of multiple sensors (e.g., wrist, ankle and waist worn accelerometers) to capture all movement is warranted among rural population with purposive sampling of all education levels.

## Background

Physical activity (PA) is a key behavioural factor for maintaining health and well-being at individual and population levels [1–3]. It has been estimated that at least 9% of premature mortality globally could be avoided if everyone adhered to the WHO physical activity guidelines [1]. Furthermore, in 2013 the economic cost to health care systems worldwide related to non-adherence was estimated at $53 billion [4]. The World Health Organization (WHO) has therefore promoted the development of PA surveillance tools in order to evaluate public health interventions and policies [5, 6] aimed at reducing the burden of non-communicable diseases (NCDs) [7].

The Global Physical Activity Questionnaire (GPAQ) is one such instrument that was endorsed by the WHO for its STEPwise Approach to Chronic Disease Risk Factor Surveillance (STEPS) [8–11]. The GPAQ was developed with special consideration of key physical activity domains in developing countries and of a length and complexity suitable for inclusion in STEPS [11].

Although GPAQ has been widely used for monitoring PA, the evidence base for its validity is limited. The most extensive study to date assessing the validity of the GPAQ was conducted in 2003–2005 in nine countries including Bangladesh [12]. However, since then an updated measure was released by the STEP wise program and evidence for the validity of the new version is still limited. Furthermore, six of these eight countries, including Bangladesh, used pedometers, a criterion measure which is not sensitive to activity intensity, and only four, of which Bangladesh was not one, included rural populations in their sample. The criterion validity and reliability of GPAQ for urban Bangladesh was found 0.06 and range 0.31–0.72, respectively [12]. Given there are substantial differences in patterns and frequency of PA between rural and urban populations [13–16], it is yet to be determined if GPAQ is an appropriate instrument to assess the status of PA among rural populations [12].

As a part of the surveillance system, nationwide surveys of NCD risk factors following the WHO STEPS strategy are being conducting periodically in Bangladesh. Comparison of three STEPS surveys’ results (2006, 2010 & 2013) of Bangladesh clearly indicated that NCDs, particularly diabetes, are increasing [17]. A comparison of the prevalence rates indicates that diabetes is becoming as problematic in rural as in urban populations [17–19]. Bangladesh is facing an escalating rise of NCDs and the validity of GPAQ for the WHO STEPS in Bangladesh needs to be established for the entire population. As the Bangladeshi sample in the validation of the previous version of the GPAQ was urban, validated against pedometers and showed very poor results, this study aimed to determine the criterion validity of the new version of GPAQ in both rural and urban populations using accelerometer as the criterion measure of physical activity.

## Methods

This study was approved by Western Sydney University Human Research Ethics Committee (HREC # H11145) and Bangladesh University of Health Sciences Ethical Review Committee.

### Participant recruitment

A total of 162 healthy participants of both genders aged 18–60 years were recruited from rural (*n* = 97) and urban (*n* = 65) areas in Bangladesh. We excluded participants with chronic medical conditions that restricted their usual activity, those with mental retardation, those who were unwilling to participate and pregnant women. We calculated our sample size to detect a Spearman correlation coefficient of 0.4 [12] as statistically significantly larger than 0 assuming a *α* = 0.05 significance level and 80% power to be *n* = 55. As we will be correlating self-reported MVPA against accelerometers in each region separately the minimal required sample was 55 urban and 55 rural (110 in total).

The rural sample was selected from Satia village of Pirganj Subdistrict of Thakurgaon District. The research assistants (RA) approached the selected households (HH), introduced the study and its importance and asked permission to enroll one eligible person from each HH. If there were more than one eligible person in a HH, study participants were chosen at random using the “last-birthday method”(i.e., the person whose birthday was last or most recent) [20]. Once a person was chosen and volunteered to participate, a date and time for data collection was arranged and the recruitment continued until the sample size reached.

For the urban sample, participants were recruited conveniently from faculty and staff of Bangladesh University of Health Sciences (BUHS), which is situated in Dhaka. There are 12 different employment grades from the highest grade (e.g., professor) to the lowest rank (e.g., cleaner). To ensure the validity study included all grades we used poster advertisements and emails to staff as well as actively approaching individual workers who were less likely to have access to email or more likely to be illiterate.

### Physical activity outcome measures

#### The global physical activity questionnaire (GPAQ)

Version 2 of GPAQ [12] in Bengali language was used in this study. GPAQ-2 collects information on the “usual/typical” week frequency (days) and duration (minutes/h) of moderate and vigorous intensity PA in three domains: 1) at work; 2) during transport; and 3) at leisure (i.e., recreational activities), comprising 16 questions in total including one question on sedentary behaviour [21]. We used the GPAQ scoring protocol [21] to create the following indicators: total MVPA MET-min and domain specific MVPA MET-min (i.e., work, transport, recreation).

METs (Metabolic Equivalent Tasks) are commonly used to express the intensity of PA. When calculating a person’s overall energy expenditure using GPAQ-2, moderate-intensity activities during work, commuting and recreation are assigned a value of 4 METs; vigorous-intensity activities are assigned a value of 8 METs. The total MVPA MET-min score is computed as the sum of all MET-min/week from MVPA performed in work, commuting and recreation.

#### Accelerometer

To investigate the criterion validity we chose the Actigraph GT3X accelerometers as objective sensor-based activity monitors to provide the criterion measure. Accelerometers are considered as more accurate than self-report for measuring time spent in different intensities and therefore used as criterion in validation of subjective self-report questionnaires [22]. The GT3X accelerometer is small, noninvasive and contains a 3-axis microelectromechanical system which measures the quantity and intensity of movement (http://actigraphcorp.com/).

Participants wore the accelerometer for seven consecutive days, except during sleep and water based activities. The device was worn at waist level above the hip of the left side. The data were stored in 10-s intervals and aggregated into 1-min epochs, a procedure recommended for accelerometer studies in adults [23]. Actigraph Actilife software was used for initialization and analyses of accelerometer data. For validity analysis, at least 10 h/day of recording were considered as a valid representative day and at least three valid days, including one weekend day, of data to represent weekly habits [23]. We compared the CV of the accelerometer MV time per week for a sample of 3 days (*n* = 155) to a sample of 4 days (*n* = 146) and we found no change in the CV (0.53 vs. 0.52). Hence for the sake of keeping the large sample we chose the lowest number of days. Atkin et al. [24] and Freedson et al. [25] cut points were taken to classify time spent in sedentary (<100 cpm), light (<1952 cpm), moderate (1952–5724 cpm), and vigorous (>5724 cpm) physical activities using vertical axis.

### Data collection

Six trained research assistants with a minimum of university graduation were recruited for data collection. All field research assistants were trained in conducting face to face interviews, including the GPAQ, and in measurements, including accelerometer data collection. Training sessions were properly guided by the facilitators and supervisors. On the first meeting day, study procedures were explained and informed consent obtained. Each participant was then fitted with an accelerometer and shown how to remove and re-wear the device. Basic socio-economic information was taken by interview on that day. A second meeting with the same interviewer was scheduled 7 days later at which the GPAQ interview was undertaken and the accelerometer collected for data downloading.

### Data analysis

After data entry, range and consistency were checked. For the general description of data, frequency analyses were calculated as number (percentage), mean (±SD) or median (IQR) when appropriate. Spearman’s correlation coefficients were used for comparison of total GPAQ MVPA MET-minutes/day, domain specific MET-minutes/day and sedentary behaviour minutes/day with accelerometer derived average minutes spent in MVPA, counts per minute, steps per day and sedentary behaviour minutes/day. Further Cohen’s Kappa statistic was used to examine the agreement of GPAQ and accelerometer in categorizing whether or not individuals meet the physical activity guidelines of at least 150 min of MVPA per week. The magnitude of bias was tested by the Bland-Altman method comparing the mean differences between MVPA MET-minutes per day from the GPAQ all domains & sedentary behaviour and accelerometers for urban and rural populations. We have presented correlations for total sample and by subgroup. Main stratification was done by place, but further stratified by gender, age and education. To interpret agreement we used following standards: 0–0.20 = poor; 0.21–0.40 = fair; 0.41–0.60 = moderate/acceptable; 0.61–0.80 = substantial; 0.81–1.0 = near perfect [12, 26]. All *p* values presented were two tailed. The statistical tests were considered significant at a level of 5% (0.05). Data was analyzed using SPSS (version23) statistical software.

## Results

The characteristics of the 162 study participants are described in Table 1. Fifty-four percent were female, the overall mean age was 35 (SD = ±9) years and 19% of the participants had no schooling, which was higher in the rural compared to the urban population (21% vs 17%). There were no significant differences in the age by gender distribution. Valid accelerometer data (i.e., ≥10 h of wear times over ≥3 days) were received from 155 participants (urban = 61, rural = 94). The mean ± SD steps/day was 9998 ± 3936 (8658 ± 2788 and 12,063 ± 4534 for rural and urban respectively, *p* = 0.0001) and the mean daily MVPA was 58 min (51 for rural and 69 for the urban, *p* = 0.001). Based on GPAQ, rural residents reported significantly higher moderate work related PA (median MET-minutes/week: 600 vs. 360 *p* = 0.02) than did urban residents.

**Table 1:** Characteristics of the study subjects according to rural and urban

Variables	Total (*n* = 162)	Rural (*n* = 97)	Urban (*n* = 65)	
	*n* (%)	*n* (%)	*n* (%)	*p* ^*1*^
Age				
≤30 years	56 (34)	29 (30)	28 (43)	0.002
31–40 years	66 (41)	38 (39)	32 (49)	
≥41years	40 (25)	30 (31)	5 (8)	
Gender				
Male	74 (46)	47 (48)	27 (41)	0.42
Female	88 (54)	50 (52)	38 (59)	
Marital status				
Unmarried	23 (14)	12 (12)	11 (17)	0.52
Married	138 (85)	84 (87)	54 (83)	
Others	1 (1)	1 (1)	0 (0)	
Education				
Illiterate	18(11)	10 (10)	8 (14)	0.0001
Informal education	13 (8)	11 (11)	2 (3)	
Primary school completed	40 (25)	31 (32)	9 (13)	
High school completed	62 (38)	39 (41)	23 (35)	
University level	29 (18)	6 (6)	23 (35)	
	Median (Q1; Q3)	Median (Q1; Q3)	Median (Q1; Q3)	*P* ^*1*^
GPAQ (MET-mins/wk)	(*n* = 155)	(*n* = 94)	(*n* = 61)	
Work				
Vigorous	0 (0; 1120)	0 (0; 1200)	0 (0; 700)	0.76
Moderate	480 (240; 1200)	600 (240;1250)	360 (42; 1020)	0.02
Total Work MVPA	840 (280; 2280)	1000 (360; 2280)	600 (130; 2390)	0.14
Travel				
Moderate	840 (480; 1680)	1060 (560;1680)	840 (420; 1680)	0.07
Recreation				
Vigorous	0 (0; 0)	0 (0; 0)	0 (0; 0)	0.94
Moderate	480 (240; 1120)	480 (270; 1140)	480 (60;1400)	0.33
Total Recreation MVPA	600 (240; 1440)	700 (300;1440)	560 (120;1680)	0.32
GPAQ Total MVPA	3320 (1680; 5760)	3440 (2270; 5880)	3220 (1180; 5710)	0.23
Sedentary mins/day	120 (60; 180)	120 (90; 180)	120 (60; 180)	0.12
	Mean (SD) Median (Q1; Q3)	Mean (SD) Median (Q1; Q3)	Mean (SD) Median (Q1; Q3)	*P* ^*2*^
Accelerometer	(*n* = 155)	(*n* = 94)	(*n* = 61)	
Valid Days	6.09 (1.38) 6 (5; 7)	5.90 (1.42) 6 (5;7)	6.38 (1.28) 6 (6; 7)	0.037
CPM (Axis 1)/day	67.42 (25.10) 64.80 (47.40; 84.90)	63.12 (21.45) 60.35 (46.95; 76.25)	74.06 (28.80) 75.20 (52.25; 93.05)	0.01
Steps/day	9998 (3936) 9082 (6969; 12,474)	8658 (2788) 8407 (6745; 10,507)	12,063 (4534) 12,353 (8509; 14,531)	0.0001
MVPA mins/day	57.96 (30.39) 50.95 (36.07; 76.00)	51.10 (25.57) 46.08 (32.87; 64.99)	68.57 (34.21) 62.03 (43.29; 92.29)	0.001
Light mins/day	211.69 (67.38) 209.30 (157.64; 253.10)	205.76 (59.09) 210.46 (150.48; 245.39)	220.83 (78.12) 207.52(158.54; 275.86)	0.20
Sedentary mins/day	551.15 (83.03) 546.03 (494.73; 607.05)	554.26 (81.43) 546.98 (497.06; 609.76)	546.35 (85.90) 546.03 (480.31; 606.12)	0.56

Results are expressed as number (%), mean (SD) and median (Q1:Q3); ns = not significant, ^1^Mann-Whitney U test; ^2^t–test

Table 2 shows the correlation between physical activity assessed by the GPAQ and measured by the accelerometer. Spearman correlation coefficients between GPAQ total MVPA and accelerometer MVPA, CPM or steps counts were low for the whole population (rho: 0.18, 0.24, and 0.28, respectively). However, stratification by place of residency indicated good correlations for urban residents (rho: 0.46, 0.55 and 0.63, respectively; *p* < 0.01) and very poor correlations for rural residents (rho: 0.0001, −0.01 and 0.05, respectively). The domain specific correlations across all indicators (i.e., MVPA, CPM or steps counts) among urban population were high for the work, travel and leisure domains (0.26 to 0.55) but among rural residents the coefficients were low for all domains. GPAQ occupational and leisure related PA showed significant fair to moderate correlation with light-intensity PA for urban population. Time in light intensity was inversely related to travel-related activity in rural area. A significant, low level of agreement between the GPAQ and accelerometer data for sitting was observed (rho: 0.23; *p* < 0.01). The agreement for categorization of participants into meeting sufficient physical activity level was fair for all participants (Kappa: 0.29; *p* < 0.0001) and good for urban participants (Kappa: 0.62; *p* < 0.0001) though low for rural participants (Kappa: 0.07) (data not shown).

**Table 2:** Spearman correlation coefficients for all GPAQ and domain specific MVPA against accelerometer indicators

Accelerometer Indicators		GPAQ				
		Total MVPA MET-mins/day	Work-related activities MET-mins/day	Travel-related activity MET-mins/day	Leisure-time activities MET-mins/day	Sitting
All	Steps/day	0.28**	0.25**	0.08	0.11	−0.14
	MVPA (mins/day)	0.18*	0.13	0.03	0.12	−0.10
	CPM (Axis 1)	0.24**	0.23**	0.02	0.11	−0.18*
	Light (mins/day)	0.17*	0.29**	−0.07	0.003	−0.21*
	Sitting (mins/day)	−0.23**	−0.21*	−0.08	−0.11	0.23**
Urban	Steps/day	0.63**	0.55**	0.52**	0.41**	−0.03
	MVPA mins/day	0.46**	0.38**	0.49**	0.26*	0.04
	CPM (Axis 1)	0.55**	0.50**	0.46**	0.29*	0.02
	Light (mins/day)	0.57**	0.58**	0.29*	0.27*	−0.10
	Sitting (mins/day)	−0.42**	−0.43**	−0.24	−0.28*	0.07
Rural	Steps/day	0.05	0.07	−0.13	−0.12	−0.17
	MVPA mins/day	0.0001	−0.03	−0.20*	0.02	−0.17
	CPM (Axis 1)	−0.01	0.02	−0.23*	−0.05	−0.33**
	Light (mins/day)	−0.2	0.05	−0.30**	−0.20	−0.27**
	Sitting (mins/day)	−0.10	−0.05	−0.001	0.02	0.38**

*Statistically significantly different from 0 at *p* < 0.05 **Statistically significantly different from 0 at *p* < 0.01

**Fig. 1:**
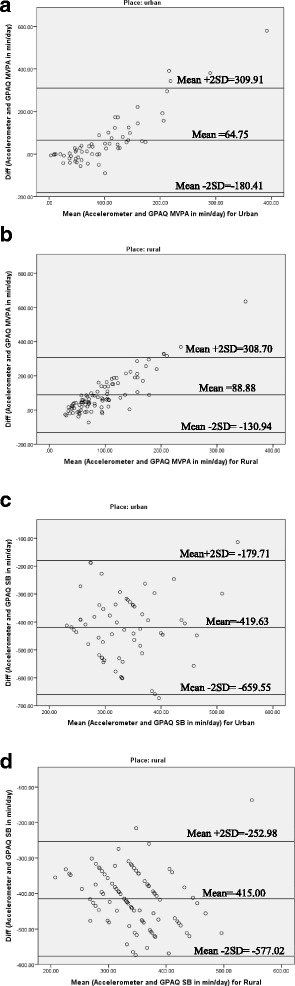
Bland-Altman plots showing the agreement between GPAQ and Accelerometer. **a**. Agreement of MVPA for urban (*n* = 61) **b**. Agreement of MVPA for rural (*n* = 94) **c**. Agreement of sedentary behaviour for urban (*n* = 61) **d**. Agreement of sedentary behaviour for rural (*n* = 94)

Figure 1a and b present the Bland and Altman plots for the agreement between GPAQ MVPA min/day for all domains and accelerometer MVPA in min/day by place. Figure 1a and b showed that the differences between the two instruments were 64.75 and 88.88 min of MVPA per day, respectively. The limits of agreement were wide with the difference lying between −180.41 to 309.91 min/day for urban and −130.94 to 308.70 for rural. A clear pattern of increased error was detected with increased average of PA. Figure 1c and d showed the difference between the two instruments in urban and rural participants were −419.63 and −415 min of SB per day with wide difference (−659.55 to −179.71 min/day and −577.02 to −252.98 min/day) which indicates negative bias exists for the GPAQ.

Table 3 shows the correlation between GPAQ total MVPA MET-mins/day and accelerometer MVPA, CPM and steps across sociodemographic subgroups. Consistent, significant correlations were found between GPAQ total MVPA and accelerometer MVPA, CPM and steps among women (rho: 0.42, 0.46 & 0.49 respectively) and young adults (age ≤35 years) (rho: 0.31, 0.32 & 0.34 respectively). After stratification by place, significant fair-to-moderate correlation was found for females, whereas urban young adults showed a significantly higher correlation than young rural adults. For education subgroups, the patterns of the correlations were inconsistent and did not follow gradient. Overall the correlations with GPAQ MVPA and accelerometer MVPA, CPM and steps were moderate for the graduate group (rho: 0.48, 0.45 & 0.51 respectively) and fair for the illiterate (rho: 0.27, 0.35 & 0.22 respectively) and primary group (rho: 0.23, 0.20 & 0.38 respectively).

**Table 3:** Spearman Correlation Coefficient between GPAQ MVPA MET-mins/day and accelerometer measures in subgroups

Sub groups		Steps/day	MVPA (mins/day)	CPM (Axis 1)
Gender				
All	Male (*n* = 70)	0.05	−0.10	0.04
	Female (*n* = 85)	0.49**	0.42**	0.46**
Urban	Male (*n* = 26)	0.56**	0.61**	0.53**
	Female (*n* = 35)	0.67**	0.55**	0.65**
Rural	Male (*n* = 44)	0.15	0.01	0.22
	Female (*n* = 50)	0.21	0.29*	0.30*
Age, years				
All	≤35 (*n* = 93)	0.34**	0.31**	0.32**
	> 35 (*n* = 62)	0.19	−0.03	0.10
Urban	≤35 (*n* = 49)	0.59**	0.48**	0.52**
	> 35 (*n* = 12)	0.73**	0.50	0.71**
Rural	≤35 (*n* = 44)	0.12	0.19	0.11
	> 35 (*n* = 50)	0	−0.18	−0.11
Education				
All	Illiterate (*n* = 30)	0.22	0.27	0.35
	Primary school level (*n* = 37)	0.38*	0.23	0.20
	High school level (*n* = 61)	0.05	−0.01	0.01
	University level (*n* = 27)	0.51*	0.48*	0.45*
Urban	Illiterate (*n* = 9)	0.30	0.12	0.53
	Primary school level (*n* = 7)	0.32	0.10	0
	High school level (*n* = 23)	0.31	0.22	0.20
	University level (*n* = 22)	0.68**	0.76**	0.67**
Rural	Illiterate (*n* = 21)	0.15	0.38	0.32
	Primary school level (*n* = 30)	0.34	0.20	0.16
	High school level (*n* = 38)	−0.28	−0.30	−0.24
	University level (*n* = 5)	0.50	0.80	0.87

*Statistically significantly different from 0 at *p* < 0.05 **Statistically significantly different from 0 at *p* < 0.01

## Discussion

To the best of our knowledge this is the first validity study of the GPAQ in Bangladesh using accelerometer and also including rural population. The results demonstrated moderate evidence of criterion related validity for total GPAQ MVPA and all domains of MVPA for urban participants but poor criterion validity for rural participants. The GPAQ demonstrated fair-to-moderate criterion validity for women, young adults (≤35 years) and those with higher level of education.

Our results contradict some findings from the nine countries validity study by Bull et al. [12]. First, the rural samples (Ethiopia, Indonesia and India) had better coefficients (rho: 0.43) than their counterpart urban sample (rho: 0.23), albeit the criterion was pedometer steps counts. Second, in the nine countries’ samples the validity coefficients were better for men than women whereas in the current study it was the other way around. Third, China and South Africa used accelerometers as the criterion measure for urban samples and the coefficient between GPAQ total PA across all domains and accelerometer moderate-intensity counts per minute were 0.24 for China sample and very poor (−0.01) for South Africa sample, much lower than the coefficient for counts per minutes in our urban sample. In the nine countries study, criterion validity for urban Bangladesh was assessed by pedometer, which is a less sensitive objective measure than the accelerometer used in the current study. The overall correlation was 0.06, which was considerably lower than in our current study where accelerometer was used [12].

Our results are comparable to other studies where low-to-moderate validity (rho: 0.20–0.48) was demonstrated against objective measures [6, 27–33]. Additionally, in the 12 countries study validation of the short International Physical Activity Questionnaire (IPAQ), the pooled validity correlations against accelerometers was found to be 0.30 (95% CI 0.23–0.36) [34]. In the Bland-Altman plot a clear pattern of increased error was detected with increased average of PA for rural and urban participants. Overestimation of GPAQ was observed in the US [27] and Singapore [28], whereas negative bias was seen in the Northern Ireland with the majority of points falling below zero [6].

Several reasons may explain the low validity of GPAQ for our rural population as opposed to the urban sample; firstly, the dominant work-related PA in rural area is farming, it is a hard work that involves digging, cutting crops, rice processing, carrying heavy loads etc. but the positioning of waist-worn accelerometers affects their ability to capture these upper body movement activities. Further, non-ambulatory activities such as cycling is also not captured by waist-worn accelerometers, and cycling is a very prevalent mode of transport in rural areas [35, 36], particularly among men. Additional explanation may be related to the pace of ambulation in the country-side which may result in accelerometer counts below the cut-off point for moderate activity [25]. For example, the Freedson determination of moderate and vigorous PA accelerometer cut points were based on walking and running on a treadmill [25] and are unlikely to capture the intensity associated with walking carrying heavy loads or on uneven surface as is common in rural areas of developing countries such as Bangladesh and thus accelerometer may underestimate total MVPA in these populations [37]. Support for this argument is the good correlation we found with time spent on light-intensity PA based on accelerometer and GPAQ occupation and travel-related physical activity, a correlation that was in the same range as for accelerometer MVPA. This may indicate that lowering the cut-point may improve the indicators for GPAQ validity.

Moreover, reference time of the GPAQ is ‘usual week’ which may create confusion in the participants to determine which particular week of a month would be best to address. The 12-countries study of validation on IPAQ discussed that the understanding of a ‘usual week’ was difficult for participants as they were not able to identify ‘what is usual?’ and participants recalled last 7 days instead of ‘usual week’ [34]. This could be more problematic if there is strong seasonal variation. In Bangladesh, there are 6 seasons and the main occupation in rural area is agriculture which follows the seasons [38]. A multi-site study of nine Asian rural areas including Bangladesh showed that PA was lower in the middle of the harvest season and increased during the more intensive harvest period [39]. Another reason for the low correlation of GPAQ MVPA in rural participants might be that accelerometer data were collected in autumn season when people are less active and GPAQ MVPA was the usual week. On the other hand urban residents had almost similar work patterns throughout the year because our urban participants were selected from one work site where occupation related PA is stable throughout the year.

In the subgroup analysis, females showed consistent correlations across all indicators of PA. In male overall correlation across indicators of PA was not seen, whereas other studies showed reverse result [6, 40, 41]. This may be due to the context specific nature of activities undertaken within both urban, and, particularly, rural Bangladesh which often requires considerable upper-body motion such as labor-intensive farming practices, as noted before, or construction jobs in the city. Moreover, males carry heavy loads such as crops, seeds, sacks etc. which limits their pace of walking. Both pedometers and accelerometers are likely to underestimate the intensity of these activities despite their being moderate-intensity efforts subjectively, as well as by energy expenditure measure (Ainsworth range 5METs to 8.5METS) [42]. In addition, swimming and cycling are common activities for rural people. Because accelerometers do not measure water-based and non-ambulatory activities, this may have contributed to the poor correlations found in males. In case of education, the patterns of the correlations were inconsistent and did not follow gradient, however, higher correlation was found in tertiary education group than other groups. This finding is similar to that of a study by Lee et al. who found that participants who had tertiary education performed better for IPAQ and over-reporting was almost double in those without tertiary education [40]. The nine countries study of GPAQ validation also showed higher correlation for those with higher education compared with those with less than 13 years of schooling [12], as in our study. Therefore it is possible that the overall low validity in rural sample was confounded by the lack of representation of participants with graduate degree in this sample. Yet, the coefficients for the illiterate groups, in both places, were better than those with primary and high-school education. The lack of gradient in coefficients by education levels suggest that factors other than cognitive errors may have contributed to the low validity such as the type of occupation they do (static, non-ambulatory).

The current study found the volume of sedentary behaviour (SB) was greater when measured by the accelerometer than by the GPAQ. This finding is similar to that of recent study where found that when SB was measured with a self-reported single item it significantly underestimates SB in comparison to accelerometer data [43]. However, a study conducted on a Chilean population found the single question from the GPAQ had fair validity for measuring SB, though poor ability for correctly classifying individuals into tertiles or quartiles of SB [30]. Our finding of a low correlation (rho: 0.23) between GPAQ measurement for minutes of sitting per day and accelerometer data agrees with previous studies [6, 12], nevertheless, this correlation coefficient increased for rural (rho: 0.38). Present findings demonstrate that GPAQ may not be appropriate when assessing minutes of SB for both urban and rural populations as it results with systematic under-estimation of amount of sitting by 7 h on average (range between 3 to 11 h) compared to accelerometer and this was true for both rural and urban population. More accurate measurement of SB may be provided by using a multiple item domain-specific questionnaire [24, 43].

The study had a number of strengths as it assessed validity of GPAQ-2 both in urban and rural population which is rare in Bangladesh and in general. Secondly, there was good compliance with accelerometer wear and adherence to the study protocol. Additionally, we followed WHO guidelines for administering the GPAQ, provided intensive training on data collection staff and close supervision during data collection to minimize avoidable sources of measurement error.

We used a triaxial Actigraph accelerometer as a reference measure for criterion validity. The gold standard measurement for assessing energy expenditure are indirect calorimetry, doubly labelled water or heart rate monitoring, however, these are expensive and require technical expertise for implementation. Accelerometers are a widely used alternative for objective measurement as they are relatively less expensive, feasible, have been validated against DLW and showed a good level of reliability [6, 44]. Nevertheless, accelerometers have their limitations. For example, in this study accelerometer data likely underestimated MVPA in the rural sample due to its inability to capture water-based, non-ambulatory and statics activities. Thus, using accelerometer as a criterion might be considered as concurrent or convergent instrument due to its pitfalls.

On the other hand, over-reporting with activity questionnaires is ubiquitous as they are prone to biases such as recall and social desirability [45, 46]. So, these could lead to overestimation of activity levels in some domains and underestimation in others. It could be better explained if we know the pattern of activities of rural Bangladesh where PA varies with seasonality. Moreover, GPAQ does not capture details of many activities culturally relevant to Bangladesh. These might be the reasons that low PA was found to be almost similar in both urban and rural (28.9 & 25.1 respectively) population in 2010 Bangladesh NCD Risk Factor Survey where GPAQ was used [47]. Hence, we suggest that in the introduction of the questionnaire the typical week should be referenced to the typical week of the season or asking about the past week, as is the case in many surveillance questionnaires. Another limitation could be that the urban sample was comprised of volunteers from a workplace setting, thus the results may not have complete reflection of the general urban population.

## Conclusion

In conclusion, the present study adds important new data on the validity of the widely-used GPAQ for estimating PA and SB levels in a low income country. For the whole population, the GPAQ performed as well as other population PA surveillance tools. Its performance with regard to the urban population was at the highest range of most surveillance tools and better than for the rural population. The GPAQ seems to be an effective tool for measuring PA in females and people with high levels of education.

Given waist worn accelerometers do not capture the typical PA in rural context; further study using a physical activity diary and a combination of multiple sensors (e.g., wrist, ankle and waist worn accelerometer) to capture all movement would be informative. Such a study should include purposive sampling of all education levels to ascertain the extent to which education level is associated with better performance.

## Abbreviations

CPM: Counts per minute
GPAQ: Global Physical Activity Questionnaire
MET: Metabolic Equivalent of Task
MVPA: Moderate to Vigorous Physical Activity
PA: Physical Activity
SB: Sedentary Behaviour
